# Fabrication and Characterization of Superhydrophobic Graphene/Titanium Dioxide Nanoparticles Composite

**DOI:** 10.3390/polym14010122

**Published:** 2021-12-30

**Authors:** Xun Hui Wu, Yoon Yee Then

**Affiliations:** 1 School of Postgraduate Studies, International Medical University, Kuala Lumpur 57000, Malaysia; 2 Department of Pharmaceutical Chemistry, School of Pharmacy, International Medical University, Kuala Lumpur 57000, Malaysia

**Keywords:** superhydrophobic, graphene, titanium dioxide, dip coating, contact angle

## Abstract

Materials with superhydrophobic surfaces have received vast attention in various industries due to their valuable properties, such as their self-cleaning and antifouling effects. These promising superhydrophobic properties are taken into high priority, particularly for medical devices and applications. The development of an ideal superhydrophobic surface is a challenging task and is constantly progressing. Various strategies have been introduced; however, a minority of them are cost-effective. This work presents a facile fabrication of the superhydrophobic surface by using graphene and titanium dioxide (TiO_2_) nanoparticles. The graphene and TiO_2_ hybrid nanoparticles are dip-coated on a biodegradable thermoplastic poly(lactic acid) (PLA) substrate. The thermoplastic PLA is approved by the Food and Drug Administration (FDA), and is widely utilized in medical devices. The graphene/TiO_2_ coating is substantiated to transform the hydrophilic PLA film into superhydrophobic biomaterials that can help to reduce hazardous medical-device complications. The surface wettability of the graphene/TiO_2_ nanoparticle-coated PLA surface was evaluated by measuring the apparent water contact angle. The surface chemical composition and surface morphology were analyzed via Fourier-transform infrared spectroscopy (FTIR) and scanning electron microscopy (SEM). The graphene/TiO_2_-coated PLA film achieved superhydrophobic properties by demonstrating a water contact angle greater than 150°. The water contact angle of the graphene/TiO_2_ coating increased along with the concentration of the nanoparticles and the ratio of TiO_2_ to graphene. Moreover, the graphene/TiO_2_ coating exhibited excellent durability, whereby the contact angle of the coated surface remained unchanged after water immersion for 24 h. The duration of the effectiveness of the superhydrophobic coating suggests its suitability for medical devices, for which a short duration of administration is involved. This study reports an easy-to-replicate and cost-effective method for fabricating superhydrophobic graphene/TiO_2_-coated surfaces, which additionally substantiates a potential solution for the manufacturing of biomaterials in the future.

## 1. Introduction

Superhydrophobic biomaterials have been commonly employed in various applications due to their non-wettability. The superhydrophobic effect of these materials is generally indicated by a high contact angle (>150°) [1,2]. The fundamental principles of superhydrophobic properties take into account the low surface energy and high surface roughness [3,4,5]. The Cassie–Baxter and Wenzel models describe the superhydrophobic wetting state attributed to the structured or patterned surface in the micro/nanoscale, which reduces direct contact between the liquid and the solid surface [6]. This surface roughness leads to water repellence, and consequently, endows the self-cleaning effect. The dirt is carried away by water as it rolls off the superhydrophobic surface [7]. In the practical situation, a transition between the Cassie–Baxter regime and the Wenzel wetting regime may occur. Nonetheless, both wetting states elucidate the wettability of superhydrophobic surfaces. In addition, the superhydrophobic surface induces an antibacterial effect by reducing the adhesion point of the microbes onto the surface. Currently, this antifouling effect is said to be an important factor for most instruments, including medical devices [1,8]. The antifouling coating can act as a fouling-resistant, fouling-release, or fouling-degrading agent [9]. Fouling-resistant and fouling-release agents prevent adhesion and weaken the bonding between the substance and the surface, whereas fouling-degrading agents destroy the adsorbed substance via an oxidizing agent or bactericidal functionalities [9,10]. In light of these mechanisms, the superhydrophobic surface is suggested to contribute as a fouling-resistant and fouling-release agent. These properties, attributed to the superhydrophobic surface, show a promising impact on the applications of medical devices.

Developments of functional superhydrophobic surfaces are mostly inspired by nature, for example, by the hierarchical structure of the lotus leaf, which provides the infamous lotus leaf effect, as well as the water-repellent surfaces of insects [11,12,13]. Various approaches to fabricating practical and functional superhydrophobic surfaces have been reviewed [9,14,15,16]. However, most of the methods are time-consuming, strenuous, and costly. These factors tend to result in low producibility. Herein, we discuss a facile, cost-effective, time-saving, and replicable strategy for fabricating superhydrophobic surfaces, namely, by dip-coating graphene and titanium dioxide (TiO_2_) nanoparticles onto a poly(lactic acid) (PLA) substrate. This method is substantiated to be employed in the large-scale production of superhydrophobic biomaterials, particularly targeting medical devices.

Graphene and TiO_2_ are introduced as the coating materials due to their tendency to improve hydrophobicity, and their high corrosion resistance and low toxicity [17,18,19,20,21]. The combination of these two materials provides a synergistic effect, lowering surface wettability via an enhancement of surface roughness. In addition to the improved self-cleaning effect, graphene/TiO_2_ nanocomposite materials exhibit enhanced antibacterial activities by suppressing the growth of both gram-negative and gram-positive bacteria via photocatalytic activity and suppressing the generation of reactive oxygen species [22,23,24,25,26]. Additionally, PLA was chosen as the substrate for coating because of its excellent mechanical properties and biodegradability, and its non-toxicity is suited to various medical applications [27]. The Food and Drug Administration (FDA) has approved the usage of PLA in, for example, surgical devices, where direct contact with biological fluids is present [28]. Previous studies substantiated that reinforcing PLA with a low concentration of nanoparticles further enhances the structural and functional properties of PLA [29,30].

The superhydrophobic graphene/TiO_2_ coating that was fabricated in this work aims to target applications, particularly medical devices, that require self-cleaning properties. The rough topography of the graphene/TiO_2_ surface provides protrusions that trap an adequate amount of air layers within the surface structure and promote resistance to bacterial adhesion. This mechanism induces the inhibition of biofilm formation [31,32]. In addition, surfaces modified with graphene and TiO_2_ have shown superior bactericidal effects and antifouling abilities [33,34,35,36,37]. The low surface energy of the modified superhydrophobic surface is substantiated to lower the cell–surface interaction, as well as the solid–liquid interaction [38,39]. The superhydrophobic surface lubricates the liquid flow by introducing an effective slip boundary, thereby generating minimum shear stress [40]. The present study offers a simple strategy for fabricating a superhydrophobic graphene/TiO_2_ coating on PLA film via the dip-coating method. Herein, we propose that the artificial superhydrophobic surface fabricated via a simple step is appropriate for imparting into medical devices, for which self-cleaning and antimicrobial properties are desirable.

## 2. Materials and Methods

### 2.1. Materials

Graphene nanoplatelets (oxidized, composition of >95% carbon and >1% oxygen, lateral dimension 2–3 µm, BET surface area 110 m^2^/g) and titanium dioxide (TiO_2_, <25 nm particle size, 99.7% trace metal basis) were obtained from Sigma–Aldrich, MO, USA. Polydimethylsiloxane (PDMS) prepolymers and curing agents were purchased from the Dow Corning Corporation, Lansing, MI, USA. Poly(lactic acid) (3052 D) was purchased from Natureworks LLC, Saint Paul, MN, USA. Chemicals, including n-hexane (C_6_H_14_, 96% purity) and chloroform (CHCl_3_), were obtained from Merck, Darmstadt, Germany, and were used as received.

### 2.2. Preparation of Thermoplastic Poly(Lactic Acid) Substrate

The thermoplastic poly(lactic acid) (PLA) film was prepared using PLA grade 3052 D. PLA pellets of 5 weight % were used to produce a transparent and resilient PLA film. The PLA pellets were dissolved in 30 mL of chloroform by stirring for 1 h at 50 °C. The clear PLA solution was cast onto a glass petri dish and dried at room temperature for 24 h.

### 2.3. Preparation of Graphene/Titanium Dioxide-Coated PLA Film

The graphene/TiO_2_ coating solution was prepared with different concentrations of nanoparticles (0.1, 0.3, 0.5, 0.7, and 0.9% *w*/*v*) in 30 mL of hexane. Each concentration of the coating solution contained graphene nanoplatelets and TiO_2_ nanoparticles with ratios of 10:0, 9:1, 7:3, 5:5, 3:7, 1:9, and 0:10. Prior to mixing, 0.3 g of PDMS (10:1 ratio of base to curing agent) was added into the solution. The suspension was then stirred for 10 min, followed by ultrasonication for 15 min. Poly(lactic acid) film (2 cm × 4.5 cm) was dip-coated with the graphene/TiO_2_ solution, at a withdrawal speed of 2 mm/s, and dried at ambient temperature. The dip-coating process was repeated 3 times. The final product was cured in the oven at 50 °C for 1 h.

### 2.4. Contact Angle Measurement

The static water contact angles on the surfaces were measured by using a goniometer (Ossila L2006A1, Ossila Ltd., Sheffield, UK). A droplet of distilled water with a volume of 10 µL was applied onto the surface of the films. The contact angle of the water droplet was measured immediately after droplet stability. The sessile water contact angles on both sides of the droplet were measured and the average value was recorded. The test was performed in triplicate.

### 2.5. Chemical Interaction and Morphological Evaluation

The surface chemical compositions were determined by using Fourier-transform infrared spectroscopy (FTIR, IRTracer-100, Shimadzu Corporation, Kyoto, Japan). The FTIR spectra were collected over a spectrum range of 400–4000 cm^−1^ with a resolution of 4 cm^−1^, and analyzed with LabSolutions IR Software (version 2.2). The surface morphology was assessed by using the scanning electron microscope (SEM, TM3000, Hitachi High-Technologies Corporation, Tokyo, Japan).

### 2.6. Durability Test

A water immersion test was used to evaluate the resistance of the graphene/TiO_2_-coated film to water exposure over a prolonged time. The graphene/TiO_2_-coated film was immersed in distilled water at room temperature for 24 h. The contact angle measurements were performed immediately after the samples were dried.

## 3. Results

### 3.1. Contact Angle Evaluation of Graphene/TiO_2_ Coating

**Figure 1:**
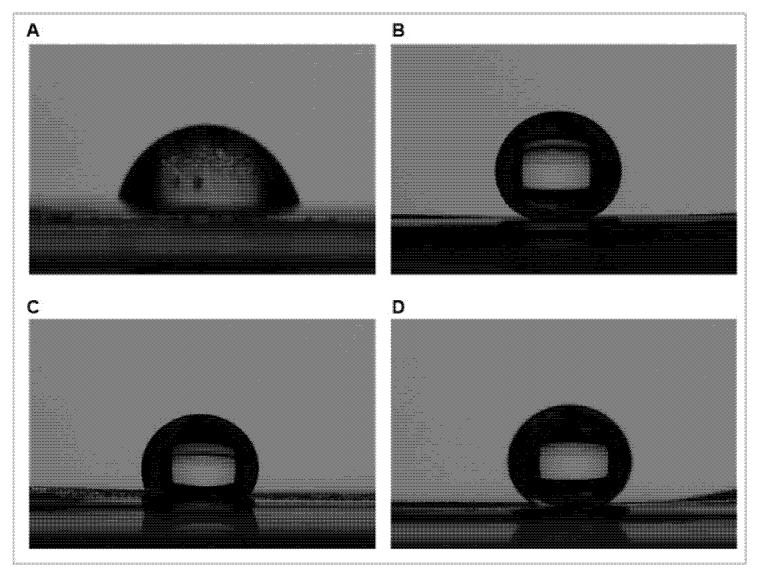
The water contact angle of poly(lactic acid) film (**A**) without the graphene/TiO_2_ coating, (**B**) with the graphene/TiO_2_ coating, (**C**) with the graphene coating, and (**D**) with the TiO_2_ coating on the surface.

The superhydrophobic graphene/TiO_2_ coating on PLA film was successfully fabricated via dip coating. The graphene/TiO_2_ nanocomposites coating achieved superhydrophobic properties, as shown in Figure 1. The low wettability of the superhydrophobic graphene/TiO_2_ is illustrated via the high apparent water contact angle. The hydrophilic PLA film, with a contact angle of 77 ± 5°, transformed into a superhydrophobic surface (contact angle of >150°) upon the application of the graphene/titanium dioxide coating.

This phenomenon suggests that the enhancement of the surface roughness by the graphene and TiO_2_ nanoparticles concomitantly improves the non-wettability of the surface. This result is in concordance with the statement mentioning that superhydrophobic properties are influenced by surface roughness and the chemical composition of the surface. In addition to the low surface energy, the graphene and TiO_2_ nanoparticles provide the hierarchical structure to the surface of PLA film and thus endow the modified surface with low wettability. The surface roughness, attributed to the micro/nano structures on the graphene/TiO_2_ surface, entrapped a sufficient air layer on the surface. Hence, high water contact angles are observed, due to the reduction of the contact point between the water droplet and the coated surface.

**Figure 2:**
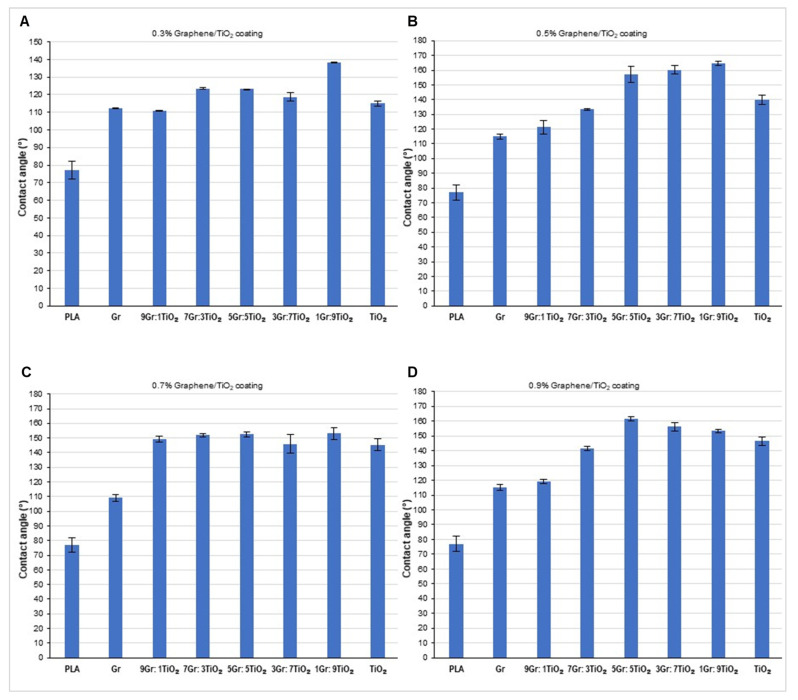
Water contact angle of the PLA film coated with (**A**) 0.3% *w/v* graphene/TiO_2_, (**B**) 0.5% *w/v* graphene/TiO_2_, (**C**) 0.7% *w/v* graphene/TiO_2_, and (**D**) 0.9% *w/v* graphene/TiO_2_. PLA without coating acts as a control; Gr represents graphene and TiO_2_ represents titanium dioxide.

The contact angle of the PLA surface increased as the concentration of the graphene and TiO_2_ coating on the surface increased from 0.1% to 0.5%. Thereafter, the contact angle of the graphene/TiO_2_-coated PLA surface slightly decreased when the concentration rose to 0.7%, followed by a surge at 0.9%. In other words, the graphene/TiO_2_ coating alters the surface structure of the PLA film and transforms the surface-wetting properties from hydrophilic to hydrophobic, and later to superhydrophobic, as the concentration of graphene/TiO_2_ nanocomposites increases (Figure 2). However, the graphene/TiO_2_ coatings exhibited a higher contact angle compared to graphene and TiO_2_ alone. This result substantiates the synergistic effect, promoting superhydrophobic properties induced by the combination of graphene and TiO_2_. It is suggested that the TiO_2_ nanoparticles covered up the empty spaces between the graphene nanoparticles, or vice versa. The mixture of these nanoparticles conferred an appropriate distance or interspacing between them. Therefore, the surface roughness established by these protrusions limits the anchoring point for the water onto the surface. In addition to acting as an adhesive agent between the nanoparticle coating and the PLA surface, the presence of PDMS lowered the surface energy of the modified surface further, and thus lowered the wettability of the graphene/TiO_2_-coated surface. In addition to lowering the surface energy, PDMS crosslinking helps to improve the elasticity and durability of the coated film [36].

It is noteworthy that, for the 0.5% *w/v* graphene/TiO_2_ coating, the water contact angle of the coated surface increased as the graphene to TiO_2_ ratio decreased. The highest contact angle, 164.21 ± 1°, was achieved with a graphene to TiO_2_ ratio of 1:9. The introduction of TiO_2_ in the coating provided an enhancement to the surface roughness by creating a rough texture on the surface. As the amount of TiO_2_ increased, the nanoparticles further increased the surface roughness by reducing the chemical inhomogeneity while maintaining the surface structure. Hence, this condition results in greater hydrophobicity [41]. However, as the ratio of graphene to TiO_2_ nanoparticles increased, the graphene nanoparticles covered the porous texture of the surface, an effect accredited to the TiO_2_ nanoparticles. The high amount of graphene nanoparticles thereby created an uneven surface with large spacings and thus lowered the contact angle [25]. The surface roughness increased when the TiO_2_ to graphene ratio was high, and vice versa. For the 0.7% *w/v* graphene/TiO_2_ coating, the water contact angle was not influenced by the graphene to TiO_2_ ratio, whereas for the 0.9% *w/v* graphene/TiO_2_ coating, the highest contact angle (161.56 ± 1°) was obtained when the ratio of graphene to TiO_2_ was equal. In concordance with previous studies [23,25], the introduction of TiO_2_ provided surface roughness to the coated surface. The addition of graphene further enhanced the textured surface by establishing the hierarchical nanostructure on the superhydrophobic surface. Therefore, both graphene and TiO_2_ exert a synergistic effect to produce a surface with superhydrophobic properties.

**Figure 3:**
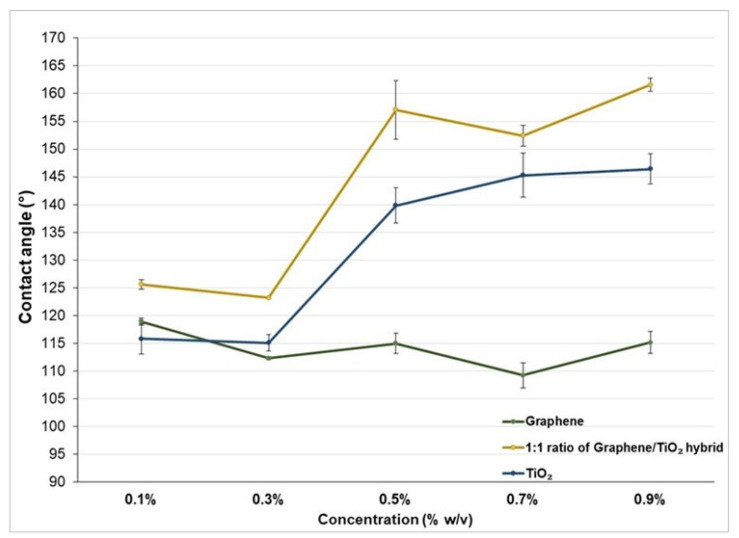
Comparison between the apparent contact angle of 0.1% *w*/*v*, 0.3% *w*/*v*, 0.5% *w*/*v*, 0.7% *w*/*v*, and 0.9% *w/v* of the graphene/TiO_2_ coating.

As the concentration of the graphene/TiO_2_ solution increased, the contact angle of the graphene/titanium dioxide-coated PLA surface increased (Figure 3). Interestingly, there was a surge in the contact angle when the concentration of graphene/TiO_2_ nanoparticles increased from 0.3% *w/v* to 0.5% *w*/*v*. This drastic increment in the contact angle was attributed to the increased amount of graphene and TiO_2_ nanoparticles; therefore, it endows sufficient surface roughness, allowing for the remodeling of the graphene/TiO_2_-coated PLA surface from a hydrophobic to a superhydrophobic surface.

The hierarchically structured surface attributed to the graphene and TiO_2_ nanoparticles allows for the entrapment of air within the contact boundary, thus reducing the contact point of the water droplet on the graphene/TiO_2_-coated surface. The porosity factor, or void ratio, was determined to estimate the volume of air entrapped by the roughened surface. The porosity factor can be calculated using the Cassie–Baxter equation [6], cos *θ*_c_ = *f_s_* (cos *θ* + 1) − 1, where *f_s_* indicates the total area of the solid–liquid interface and *θ*_c_ represents the contact angle of the coated PLA surface, whereas *θ* represents the contact angle of the smooth PLA surface. By substituting 77°, which is the contact angle of the smooth unmodified PLA film, and the contact angles of the respective coated surface into the equation, the result for *f_s_* can be determined (Table 1). The result shows that the *f_s_* value for the superhydrophobic surface is less than 0.1, which is in agreement with the previous studies reported by Karapanagiotis et al. and Basu et al. [42,43] The surface roughness attributed to the graphene and TiO_2_ allowed for a sufficient amount of air entrapment on the surface, thereby lowering the wettability and endowing the graphene/TiO_2_-coated surface with superhydrophobic properties.

### 3.2. Surface Chemical Composition and Surface Morphology Evaluation

**Figure 4:**
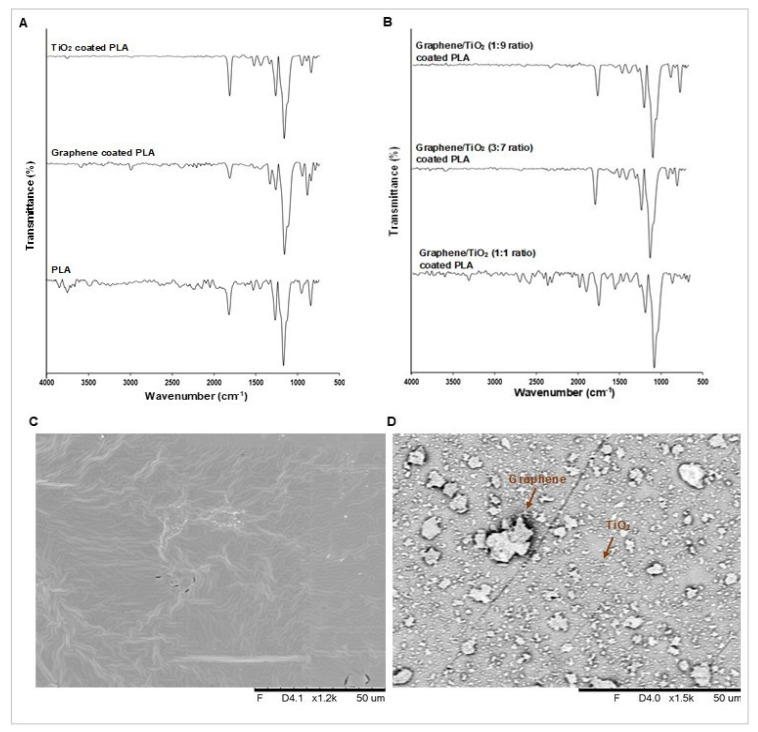
FTIR spectra of (**A**) the PLA film without coating, the graphene-coated film, and the TiO_2_-coated film, and (**B**) the graphene/TiO_2_-coated film. SEM images of (**C**) the PLA film and (**D**) the graphene/TiO_2_-coated PLA film.

The chemical functional groups of the graphene/TiO_2_ coating are characterized via FTIR, as illustrated in Figure 4. The peaks between 3000 cm^−1^ to 3600 cm^−1^ represent the O−H stretching. The peaks around 1000 to 1200 cm^−1^ represent the C–O−C stretching of PLA. The peaks of ester are depicted at 1083 cm^−1^, belonging to the C–O−O stretching vibration, and circa 3000 cm^−1^, which belongs to the CH_3_ stretching band. Moreover, the asymmetric and symmetric C−H deformations in CH_3_ bending are depicted by the peaks at 1454 cm^−1^ and 1365 cm^−1^. The carbonyl C=O stretching is depicted as the peak at 1751 cm^−1^. The bands between 1630 cm^−1^ to 1800 cm^−1^ represent the C=C bond [44,45,46,47]. The presence of TiO_2_ produces an intensified peak at 1454 cm^−1^. The Ti−O stretching band can also be observed at 890 cm^−1^ and 750 cm^−1^ [18,48] The peaks for PDMS are observed at 860 cm^−1^ and 1253 cm^−1^, attributed to the in-plane and out-of-plane bending of the Si−CH_3_ [25]. The peaks between 2900 cm^−1^ and 3000 cm^−1^ represent C−H stretching, while the peaks between 1000 to 1400 cm^−1^ represent C−O stretching. The peaks around 1000 cm^−1^ to 1130 cm^−1^, which represent the Si–O–Si bands of PDMS, is suggested to be overlapped with the C−O-stretching bands.

**Figure 5:**
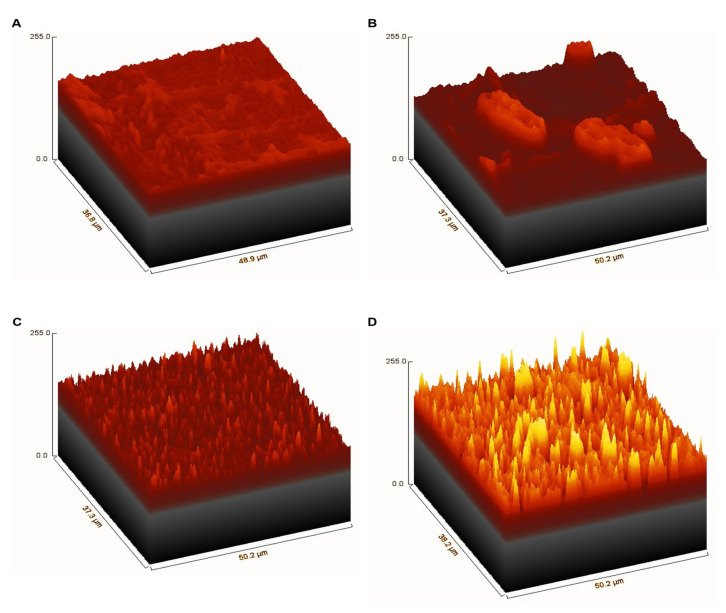
Three-dimensional representation of SEM images of (**A**) the non-coated PLA film, (**B**) the graphene-coated film, (**C**) the TiO_2_-coated film, and (**D**) the graphene/TiO_2_-coated film.

The surface morphologies of the non-coated PLA film and the graphene/TiO_2_-coated film are illustrated in Figure 4 and Figure 5. Undoubtedly, PLA exhibits a smooth surface. The surface morphology of the graphene/TiO_2_-coated surface shows a homogenous distribution of graphene and TiO_2_ nanoparticles. The uniform coverage of graphene and TiO_2_ nanoparticles provides a roughness to the surface, which is accountable for the low surface wettability. The graphene nanoplatelets are irregular in shape, whereas the TiO_2_ nanoparticles appear to have a nanoscopic particle size of less than 50 µm. The hierarchical structure on the coated surface is attributed to the graphene/TiO_2_ nanoparticles acting as protrusions. The interspacing between the protrusions allows for an adequate amount of air pockets to be entrapped on the surface. As depicted in the 3D images in Figure 5, the surface roughness can be estimated via the presence of protrusions on the coated surface. The graphene/TiO_2_ coating provides protrusions with different heights and interspacing, which help to minimize the contact point of liquid on the surface. This result correlates with the aforementioned porosity factor in Table 1. The porosity factor of the surface or the total surface area of the solid–liquid interface decreases drastically upon the introduction of graphene and TiO_2_ nanoparticles. The graphene/TiO_2_ hybrid nanocomposite confers a higher degree of surface roughness, therefore manifesting a greater reduction in the total surface area of the solid–liquid interface. The mixture of graphene and TiO_2_ nanoparticles offers an optimal surface roughness, which allows for a sufficient amount of air-layer entrapment, thus furnishing the coated surface with superhydrophobic properties. We suggest that the Cassie–Baxter regime be used for this scenario. The minimal solid–liquid interface, consequently, results in the high-water contact angle, and hence, the superhydrophobic properties.

### 3.3. Durability Test

**Figure 6:**
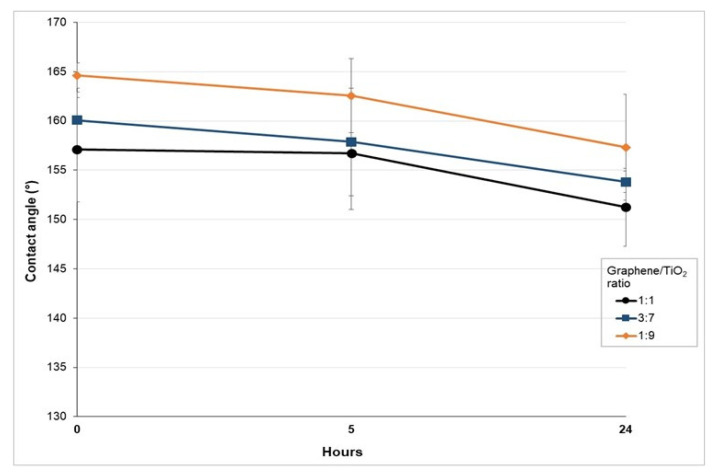
The durability of the superhydrophobic graphene/TiO_2_-coated films via contact-angle measurement upon water immersion for 5 h and 24 h.

The durability of the graphene/TiO_2_-coated films are evaluated via the water immersion method, according to ASTM D870 [49]. The results from the water immersion test substantiated the excellent durability and stability of the graphene/TiO_2_-coated film (Figure 6). The contact angles of the graphene/TiO_2_-coated film decreased by approximately 2° after being immersed in water for 5 h. An additional period of water immersion, up to 24 h, further decreased the contact angles by approximately 5°. Nonetheless, the contact angles of the three different ratios of the graphene/TiO_2_-coated films remained above 150° at the end of the water immersion test. This indicates that the immersion of graphene/TiO_2_-coated film in water for a prolonged period does not result in a significant degradation of the coating. In the practical setting, it is not uncommon for medical devices to be in direct contact with body fluids for less than 8 h. The result validates the effectiveness of the graphene/TiO_2_-coated surface to be utilized for ample time. Hence, this study asserts the durability and robustness of the superhydrophobic graphene/TiO_2_-coated PLA film and substantiate it to remain functional in the long run.

The PLA films demonstrated superhydrophobic properties upon modification with the graphene/TiO_2_ nanoparticles composite. This study reports the effectiveness of the graphene/TiO_2_ nanoparticles for fabricating the superhydrophobic surface. The water-repelling effect exhibited by the graphene/TiO_2_ coating shows a promising antifouling and self-cleaning effect. The strategy herein is proposed as an ideal manufacturing method for superhydrophobic surfaces that are suitable for employment in medical devices. Further investigation is required to study the antibacterial activity, as well as the cytocompatibility, of the graphene/TiO_2_-coated PLA surface. Nonetheless, this study offers a simple, time-saving, and eco-friendly method of fabricating a durable superhydrophobic graphene/TiO_2_-coated surface.

## 4. Conclusions

A superhydrophobic surface was successfully fabricated by using graphene and TiO_2_ with a minimal concentration of 0.5% *w*/*v*, via a simple dip-coating method. The graphene/TiO_2_ coating showed superhydrophobic properties by exhibiting a high apparent contact angle. The apparent contact angle increased with the concentration of nanoparticles. Moreover, the coating of graphene and TiO_2_ hybrid exerted a synergistic effect by providing an enhanced surface roughness on the PLA surface. It is implied that the structured surface allowed for the entrapment of air pockets, which reduced the direct contact point between the water and graphene/TiO_2_-coated surface. Therefore, the graphene/TiO_2_-coated PLA film demonstrated low wettability. Additionally, the ratio of graphene to TiO_2_ influenced the wettability of the coated surface. In brief, a superhydrophobic surface was achieved by using a minimal amount of graphene and TiO_2_ nanoparticles. In addition, the graphene/TiO_2_ coating possesses significant durability and stability by demonstrating low degradation after overnight water immersion. This study shows a promising nanoparticles composite, prepared with graphene and TiO_2_, that is suitable for introduction into medical devices, where they can deploy their antifouling properties.

## Figures and Tables

**Table 1:** The total area of the solid–liquid interface of the graphene/TiO_2_-coated PLA film with the concentration of 0.1% to 0.9% and different graphene to TiO_2_ ratios. Gr and TiO_2_ represent the abbreviation of graphene and titanium dioxide, respectively.

	Total Area of Solid–Liquid Interface, *f_s_*						
	Gr Only	Gr/TiO_2_ (9:1)	Gr/TiO_2_ (7:3)	Gr/TiO_2_ (1:1)	Gr/TiO_2_ (3:7)	Gr/TiO_2_ (1:9)	TiO_2_ Only
0.1% *w/v* Gr/TiO_2_	0.42	0.38	0.38	0.34	0.40	0.39	0.46
0.3% *w/v* Gr/TiO_2_	0.51	0.53	0.36	0.37	0.42	0.21	0.47
0.5% *w/v* Gr/TiO_2_	0.47	0.39	0.26	0.06	0.05	0.03	0.19
0.7% *w/v* Gr/TiO_2_	0.55	0.11	0.10	0.09	0.14	0.09	0.15
0.9% *w/v* Gr/TiO_2_	0.47	0.42	0.18	0.04	0.07	0.09	0.14
